# 5-Hydroxymethylcytosine profilings in circulating cell-free DNA as diagnostic biomarkers for DLBCL

**DOI:** 10.3389/fcell.2024.1387959

**Published:** 2024-11-15

**Authors:** Hangyu Chen, Maimaitiyasen Duolikun, Hai-Chuan Zhu

**Affiliations:** ^1^ Institute of Biology and Medicine, College of Life and Health Sciences, Wuhan University of Science and Technology, Wuhan, China; ^2^ Department of Pharmacy, Peking University Third Hospital, Beijing, China; ^3^ Key Laboratory of Tropical Biological Resources of Ministry of Education, School of Pharmaceutical Sciences, Hainan University, Haikou, China

**Keywords:** epigenetics, DLBCL, 5-hydroxymethylcytosine (5hmC), logistic regression modeling, cell-free DNA

## Abstract

**Background:**

5-Hydroxymethylcytosine (5hmC) is an important DNA epigenetic modification that plays a vital role in tumorigenesis, progression and prognosis. Previous studies have shown that it plays an important role in the prognosis of diffuse large B-cell lymphoma (DLBCL) and in the prediction of the efficacy of R-CHOP therapy. However, its potential for diagnosing DLBCL has not been reported. Here, we investigated the utility of 5hmC in plasma cfDNA in the diagnosis of DLBCL.

**Methods:**

Applying 5hmC-Seal technique, we obtained genome-wide 5hmC profiles in plasma cell-free DNA (cfDNA) samples from 176 Chinese subjects, included 86 DLBCL patients and 90 healthy controls. To investigate whether 5hmC can be used as a diagnostic biomarker for DLBCL, we separated patients and healthy controls into training (DLBCL = 56, Healthy = 60) and validation (DLBCL = 30, Healthy = 30) cohorts and developed a 5hmC-based logistic regression model from the training cohort to diagnose the DLBCL patients in the validation cohort.

**Results:**

In this study, we found 10 5hmC biomarkers, and the models created by these differentially regulated 5hmC modified genes showed high accuracy in distinguishing DLBCL patients from healthy controls (validation cohort: AUC = 0.94; (95% CI 88.8%–99.4%)).

**Conclusion:**

Our study suggested that 5hmC markers derived from plasma cfDNA can served as effective epigenetic biomarkers for minimally invasive diagnosis of DLBCL.

## Introduction

Diffuse Large B-Cell Lymphoma (DLBCL) is an aggressive cancer, accounts for about 30% of all lymphomas (Sehn and Salles, 2021), and it is estimated that there are approximately 150,000 new cases of DLBCL worldwide each year (Sehn and Salles, 2021). Currently, the diagnosis of DLBCL is mainly based on biopsy and puncture of the lesion site tissue (Malpica et al., 2022). However, tissue biopsies cannot be repeated, and the results of pathological analysis are affected by tumor heterogeneity. Therefore, discovering a set of noninvasive surrogate markers that diagnose DLBCL is urgently needed.

Recently, considerable attention has been focused on the modification of 5hmC in cell-free circulating DNA (cfDNA). This modification has gained significant interest as it offers a non-invasive approach for diagnosing and predicting human diseases through liquid biopsies (Zemmour et al., 2018; Luo et al., 2021). It is well known that cfDNA is endogenous DNA free from cells and released into blood and other body fluids through apoptosis or necrosis (Yeh et al., 2017; Tuchalska-Czuroń et al., 2020). In addition, tumor cells release DNA into serum or plasma, enable detection of cancer-associated genetic alterations (Diaz and Bardelli, 2014). cfDNA fragmentomics analysis studies have shown that the length of cancer-derived cfDNA may be more variable than that of cfDNA from non-cancer cells, and that these differences reflect changes in chromatin structure and other genomic and epigenome abnormalities in cancer. cfDNA fragments can be used as biomarkers for cancer detection in a location-specific manner (Snyder et al., 2016; Cristiano et al., 2019; An et al., 2023). At the same time, some studies have proved that the cfDNA fragment pattern is related to the cfDNA epigenetic pattern, which can be used as a marker for cancer detection in combination (Zhou et al., 2022). Several recent studies have reported that somatic mutations reflecting changes in primary tumor genes can be detected in cfDNA of DLBCL patients. Additionally, abnormal promoter methylation of acellular circulating DNA has been observed in the plasma of DLBCL patients (Bohers et al., 2015; Kristensen et al., 2016). Thus detecting genetic and epigenetic biomarkers in cfDNA has emerged as a promising noninvasive approach for the diagnosis, prognosis, and treatment of cancer (Li W. et al., 2017; Lo et al., 2021; Luo et al., 2021). Epigenetic changes play a major role in both normal B cell maturation and DLBCL development (Wedge et al., 2017).

Specifically, 5-methylcytosine (5 mC), as a fundamental component of DNA methylation, has shown promising potential for diagnosis and therapy in various critical areas such as prenatal testing, oncology, and transplantation monitoring (Sun et al., 2015; Loyfer et al., 2023). With the advancement of research, there has been a growing interest in the scientific community regarding 5-hydroxymethylcytosine (5hmC), which is the oxidation product of 5 mC and is catalyzed by the 10–11 translocation protein family. It is not only considered to be a relatively stable active DNA demethylation intermediate, but also regarded as a novel epigenetic marker of cancer (Vasanthakumar and Godley, 2015; Chen et al., 2016). Recent studies have shown that 5hmC patterns in cfDNA plays a critical role in gene expression regulation, as well as in the carcinogenesis of multiple solid tumors (Li W. et al., 2017; Song et al., 2017). Moreover, the role of 5hmC in prognosis of DLBCL and its response to R-CHOP treatment has been extensively investigated (Chiu et al., 2019; Chen et al., 2021), but its potential in the early diagnosis of DLBCL remains largely unexplored. Furthermore, exploring the diagnostic value of 5hmC in DLBCL can contribute to more accurate and timely identification of this disease. Therefore, 5hmC in cfDNA have potential to be promising biomarkers for minimally invasive diagnosis in DLBCL.

In this study, we used 5hmC-Seal technique to obtain genome-wide 5hmC profiles from plasma cfDNA of 86 DLBCL patients and 90 healthy controls. Our results demonstrated that DLBCL patients and healthy controls had distinct 5hmC profiles and that 5hmC markers selected by machine learning algorithms may serve as preliminary research for the diagnosis of DLBCL and provided new insight for the future molecularly target therapy of DLBCL.

## Materials and methods

### Study participants

In total, 86 DLBCL patients were enrolled from the multi center studies including Peking University Third Hospital, Fifth Medical Center of PLA General Hospital, and Cancer Hospital Chinese Academy of Medical Sciences, from 2017 to 2019. All patients had signed the patient consent form. In all cases, the diagnosis of DLBCL was made using appropriate diagnostic criteria from the 2016 WHO classification of lymphoid tumors with combinations of histologic, immunohistochemical, and cell of origin (Coo) defined according to the Hans algorithm (Fang et al., 2010). This study was conducted in accordance with the Declaration of Helsinki.

### Clinical samples collection and cfDNA isolation

Peripheral blood samples (8–10 mL) from DLBCL patients were obtained through routine intravenous blood sampling and collected into a Cell-Free DNA collection tube (Roche). Plasma separation was performed within 24 h, Whole blood samples were centrifugation twice at 4°C 1350×g for 12 min and once at 4°C 13,500×g for 12 min. The plasma layers were then transferred to a new tube. Then the plasma samples were immediately stored at −80°C for future use. The plasma cfDNA was extracted from 2–4 mL plasma using the Quick-cfDNA Serum & Plasma Kit (ZYMO) and then stored at −80 °C. The concentration and quality of cfDNA were quantified by Qubit fluorometer and nucleic acid electrophoresis before library preparation.

### 5hmC library construction and high-throughput sequencing

5hmC libraries for all cfDNA samples were constructed using the high-efficiency hmC-Seal technology described previously (Song et al., 2011). Given the high sensitivity of the chemical labeling method, we assigned low values of 1–10 ng for the input cfDNA. The plasma-derived cfDNA was subjected to end-repairing and then ligated with sequencing compatible adaptors. 5hmC bases were biotinylated via two-step chemistry and purified by the DNA Clean & Concentrator Kit (ZYMO), and subsequently enriched by binding to Streptavidin beads (Life Technologies). Then, the beads were re-suspended in RNase-free water and amplified with 14–16 cycles of PCR amplification. Finally, the PCR products were purified using AMPure XP beads (Beckman). All libraries were quantified with a Qubit 3.0 fluorometer (Life Technologies). 5hmC sequencing was performed on the Next-Seq 500 platform according to pairedend 38-bp high-throughput sequencing. Finally, we used the Agilent 2,100 bioanalyzer for quality control of the 5hmC library and based on the strip size to determine the presence of adaptor dimers (120-150bp).

### Mapping and identifying 5hmC-enriched regions

FastQC (version 0.11.5) was used to check the sequence quality. Additionally, bowtie2 (version 2.2.9) was adopted for aligning raw reads to the human genome (version hg19) (Langmead and Salzberg, 2012), and then filtered with SAM tools (version 1.9) (parameters used: Sam tools view-f 2-F 1548 -q 30) (Li et al., 2009). Subsequently, the paired-end reads were converted into the Bed Graph format, and normalized them to the overall quantity of aligned reads by exploiting bed tools (version 2.19.1) (Langmead, 2010). Meanwhile, with the aid of UCSC BedGraphToBigWig, we also converted the paired-end reads into the BigWig format, so that the Integrated Genomics Viewer-assisted visualization could be achieved. Potential 5hmC enriched regions were identified using MACS2 (version 2.1.1) in each sample (Zhang et al., 2008). Peak regions that appeared in more than 10 samples and that were less than 1,000 bp were combined into one unified catalog for each patient. Genomic regions that tend to show artifact signals, according to ENCODE, were filtered out. The 5hmC enriched regions were generated by intersecting the individual peak call file with the merged peak file. We annotated the 5hmC-enriched region using the CHIP seeker package and used the genes closest to this region for subsequent analysis.

### Feature selection, model training, and validation

DLBCL patients were randomly divided into training and validation groups in a ratio of 2:1; using train_test_split in scikit-learn (version 0.22.1) package in Python (version 3.6.10), the logistic regression CV (LR) model was chosen to establish warning models (Abraham et al., 2014). In the training cohort, we identified deferentially 5hMc-enriched regions (DhMRs) using DESeq2 package (version 1.30.0) in R (version 3.5.0), with the filtering threshold (*p*-value <0.05 and |log2FoldChange| ≥ 0.5). To avoid overfitting, 10-fold cross-validation was carried out for 5 rounds in the following manner. Subsequently, this study carried out 100 repeats for further filtering with the use of Scikit-Learn module’s recursive feature elimination (parameters adopted: estimator = LogisticRegressionCV (class weight = “balanced”, cv = 2, max_iter = 1,000), scoring = “accuracy”). Meanwhile, tenfold cross-validation was repeated 100 times in each round, and the final markers observed in at least three rounds were used to build the final warning model in the training cohort. Next, trained LR model was used to predict the treatment outcome for patients in the validation cohort. Receiver operating characteristics (ROC) analysis was used to evaluate model performance. Area under the curve (AUC), best cutoff point, sensitivity, and specificity were computed with sklearn metrics module. A weighted diagnostic score (wd-score) was then calculated as the sum of the gene-wise product of logistic model coefficients and corresponding 5hmC marker value for each individual: wd-score = $\sum_{k=1}^{n}\beta k\times genek$ , where β _k_ is the coefficient from the final multivariable logistic model for the *k*th marker gene, and *gene*_*k*_ is the 5hmC level of the *k*th marker gene. The area under curve (AUC) and 95% CIs were generated to evaluate the model performance.

### Exploring the functional relevance of the 5hmC modified genes

Differentially hydroxymethylated genes (DhMGs) were annotated using R Package’s ChIPseeker 1.20.0 (Schmitz et al., 2018), and further functional assessments were accomplished on the genes situated nearest to the marker zones. The enrichment analysis of the GO biological process (BP) was completed by the ClueGO (version 2.5.8) and CluePedia (version 1.5.8) plug-in from Cytoscape software (version 3.7.1). Additionally, medium network specificity, Bonferroni adjusted *p* < 0.01, and enriched gene number >5 were chosen as the criteria for significance. We used the Search Tool for the Retrieval of Interacting Genes (STRING) database (version 10.0, https://string-db.org) to find protein–protein interactions for 5hmC markers. Then, the Cytoscape software was used to construct the PPI network.

### Survival analysis and gene expression correlation analysis in TCGA-DLBC

For our survival analysis, we utilized the gdc-client (version 1.5.0) to download mRNA HTseq FPKM data from 48 patients with Diffuse Large B-Cell Lymphoma (DLBCL) as part of the TCGA-DLBC dataset (Li T. et al., 2017) from the GDC Data Portal. Concurrently, we manually retrieved curated clinical data, encompassing overall survival (OS), disease-specific survival (DSS), disease-free interval (DFI), and progression-free interval (PFI), from the UCSC Xena platform (Goldman et al., 2020). The survival analysis in this study utilized the Survminer package (version 0.4.6) and Surviva packages (version 2.44–1.1) in R. Forty-eight patients were categorized into either the high-expression group or low-expression group based on cutoff points determined by the maximally selected rank statistics algorithm. Survival analysis for each gene was conducted using Kaplan-Meier curves (Barakat et al., 2019) and the log-rank test (Torbicki et al., 2019). For the survival analysis, *p*-value <0.05 was considered statistically significant. For gene expression correlation analysis, we used a web tool called TIMER2.0 (Li et al., 2019), which incorporated all TCGA expression data, to explore the mRNA expression relationship between 5hmC markers and other genes of interests in the TCGA DLBC dataset. The correlation analysis was done using Spearman rank correlation.

### Statistical analysis

With the use of GraphPad Prism 8, data were statistically processed as detailed in Table 1. For data showing normal distribution, two-tailed t-tests (either paired or unpaired) were employed. With the purpose of calculating 95% confidence intervals, the percentile method was used. Differences were thought to be of significance with *p* < 0.05.

**TABLE 1:** characteristics of healthy controls and patients with DLBCL.

Characteristics	Subgroup	Whole set (n = 86)	Training set (n = 56)	Validation set (n = 30)
Age (patients/healthy)	Patients with DLBCL	86	56	30
	<40	20/57	15/38	5/19
	40–49	7/19	3/11	4/8
	50–59	25/11	15/9	10/2
	≥60	34/3	23/2	11/1
	Mean (SD)	54.7 (15.5)	54.4 (16.2)	55.2 (14.4)
Sex (patients/healthy)	F	30/36	22/25	18/11
	M	46/54	34/35	12/19
Stage	I	6	2	4
	II	18	11	7
	III	7	4	3
	IV	48	33	15
	Unknown	7	6	1
Subtype	BCL2-	18	10	8
	BCL2+	50	34	16
	Unknown	18	12	6
	Mean LDH (SD)	363.33 (362.72)	362.95 (361.87)	364.04 (363.44)
	Meanβ2MG (SD)	2.84 (2.77)	2.86 (2.73)	2.80 (2.84)

LDH, actate dehydrogenase; β2MG, beta2 microglobulin.

## Results

### Clinical characteristics of diffuse large B Cell lymphoma (DLBCL) patients

Plasma samples from 86 DLBCL patients (Male 46, Female 40), and 90 healthy donors were collected. Clinical data were collected from all samples, and detailed information is listed in Table 1. The mean age of all patients was 54.6 years. Besides, there were 50 BCL2-positive patients, 18 BCL2-negative patients and 18 unknown patients (2.3%). Finally, the mean values of LDH and β2MG in all patients were 364.33 U.L^−1^ and 2.84 mg.L^−1^, respectively.

### 5hmC profiles differ between healthy volunteers and DLBCL patients

**FIGURE 1:**
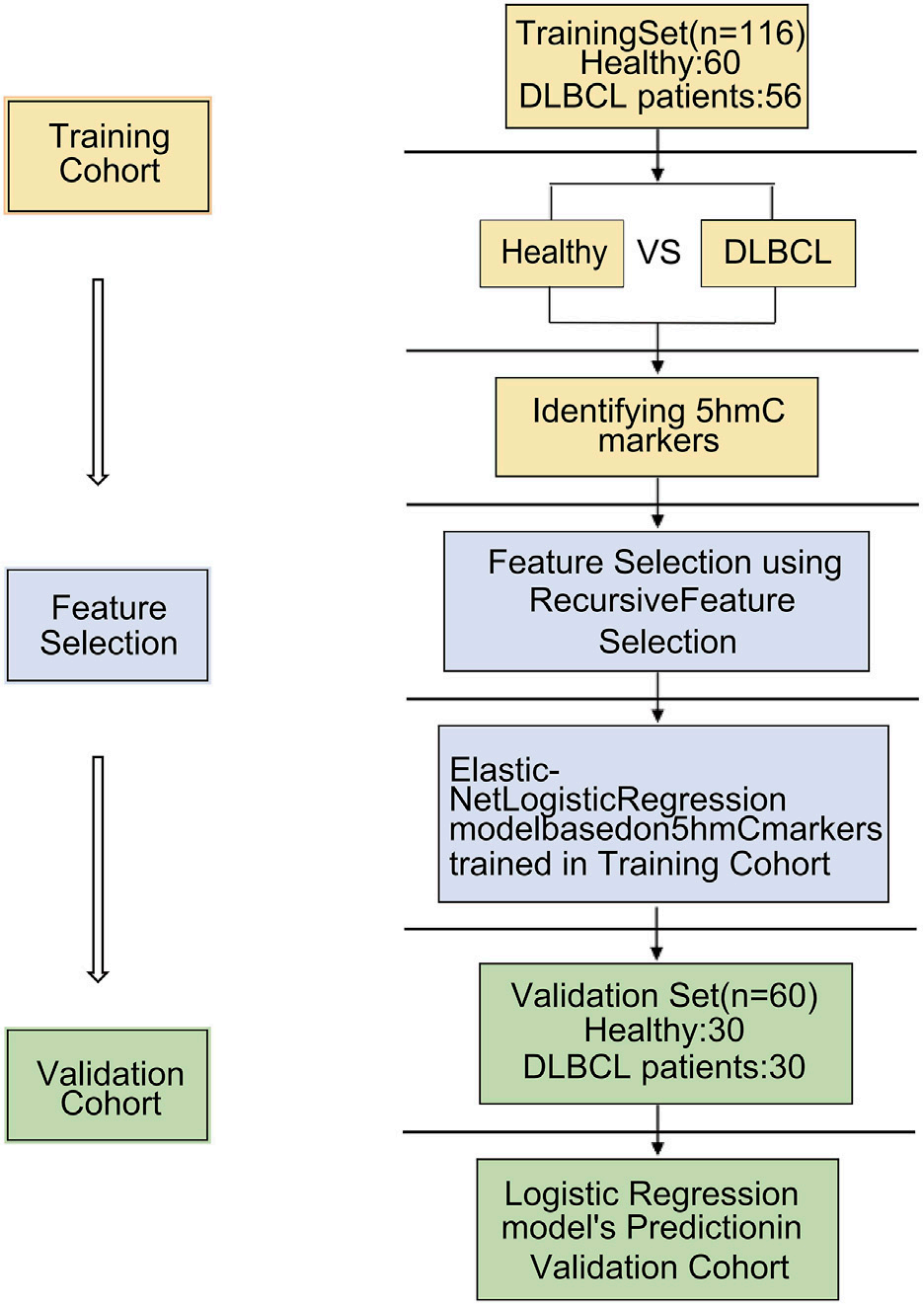
Overview of study design. A total of 86 cfDNA samples were collected at the time of diagnosis from patients with DLBCL. 86 DLBCL patients and 90 healthy controls were randomly divided into a training cohort and validation cohort. A logistic regression model was trained by the training cohort that was used to predict treatment response in the validation cohort.

**FIGURE 2:**
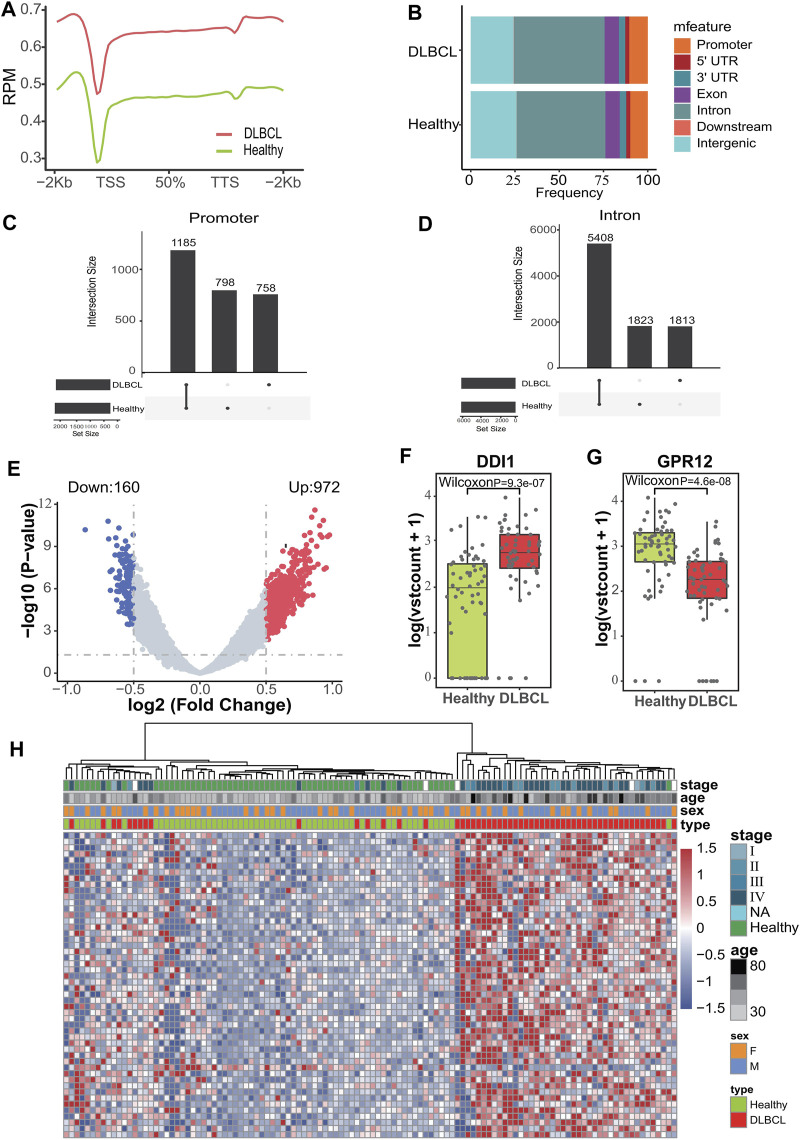
Characteristics of 5hmC distribution in plasma cfDNA of DLBCL patients. **(A)**, The profiled 5hmC-Seal data in all samples cfDNA are enriched in gene bodies and depleted in the flanking regions. **(B)**, Presence of 5hmC peaks that overlapped within biological replicates on different genomic elements. **(C,D)**, Venn diagram showing gene number associated with peaks observed of those two groups. **(E)**, Volcano plot. Significantly altered DhMGs (|log2FC| > 0.5, *p*-value < 0.05) are highlighted in red (up) or green (down) using the DLBCL patients vs. Healthy controls cfDNA samples. Grey dots represent the genes that are not differentially expressed. **(F,G)**, Boxplots of *DDI1* and *GPR12*. Log2 transformed of TMM normalized 5hmC enrichment values were plotted, and the Wilcoxon *t*-test was used. **(H)**, Heatmap of top 50 DhMGs with sample type, age, and sex information labeled. Unsupervised hierarchical clustering was performed across genes and samples.

86 DLBCL patients and 90 healthy volunteers were randomly divided into a training cohort (DLBCL = 56, healthy = 60) and validation cohort (DLBCL = 30, healthy = 30) (Figure 1). First, 5hmC-Seal was performed with extracted cfDNA to map the genome-wide 5hmC profiles for all samples. In training cohort, sequencing data showed that 5hmC was mainly enriched at transcription start sites (TSS) and transcription end sites (TTS) (Figure 2A), which was consistent with previous reports (Quail and Joyce, 2013), suggesting that the accumulation of 5hmC is related to transcriptional activity. 5hmC was mostly distributed (75%) on the gene body (GB) in the four groups, and the relative enrichment of 5hmC on GBs was the highest in the DLBCL group (Figure 2B). With the aim of increasing the significance of the findings, our study employed highly stringent peak selection criteria and selected peak base pairs that overlapped in the biological replicates (Figures 2C,D; Supplementary Figure S1A). As a result, during disease development, 5hmC loss is attributed to the intergenic regions and tends to accumulate slowly on GBs. Differential analysis between healthy volunteers and DLBCL patients showed that there were 972 genes with high hydroxymethylation and 160 genes with low hydroxymethylation in DLBCL (Figure 2E, Supplementary Table S1). For instance, *DDI1* (Figure 2F) was highly enriched in hydroxymethylation for DLBCL (*p* = 9.3e-07), and *GPR12* (Figure 2G) was highly enriched in hydroxymethylation for healthy volunteers (*p* = 4.6e-08). Finally, using the default clustering methods, the heat map results showed that Top50 DhMRs in these 1,132 DhMRs could effectively separate DLBCL patients from healthy controls (Figure 2H).

### Pathway analysis and function exploration

**FIGURE 3:**
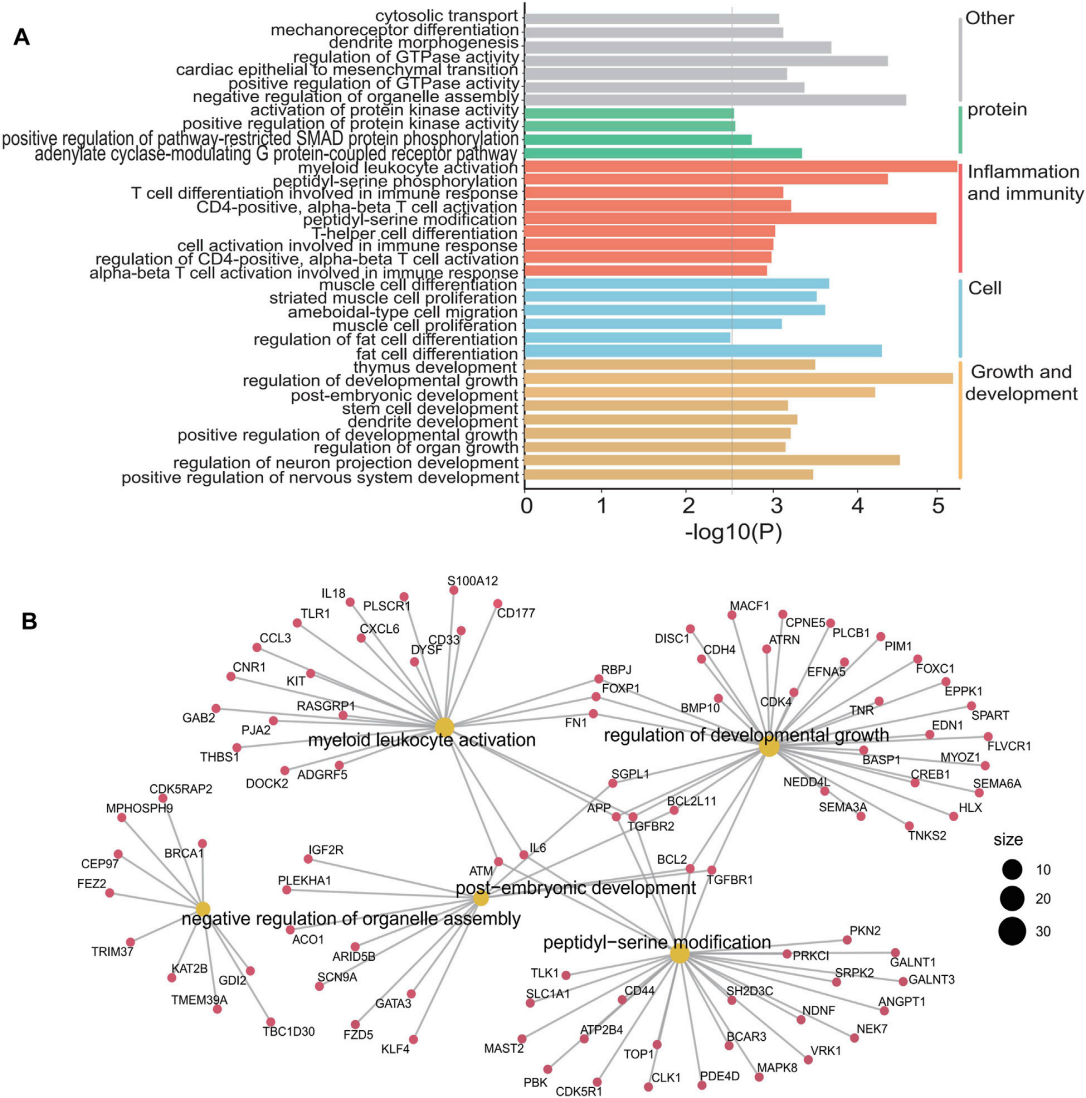
GO enrichment analysis and function exploration of 1,132 5hmC markers using Cytoscape software. **(A)**, GO enrichment bar plot. **(B)**, GO enrichment and Gene-Concept Network.

Pathway analysis of 1,132 differentially hydroxymethylated genes (DhMGs) (Supplementary Table S1) in DLBCL patients suggested functional enrichment in certain canonical pathways. The GO biological pathways is mainly concentrated in immune and inflammation related signaling pathways such as myeloid leukocyte activation, CD4-positive alpha-beta T cell activation, peptidyl-serine modification, T cell differentiation involved in immune response and cell activation involved in immune response (Figure 3A). Among these pathways, signaling by alpha-beta T cell differentiation was known to be relevant to tumor growth and apoptosis, which suggested that the DhMRs might be involved in the immunity system (Aucamp et al., 2018; Cioroianu et al., 2019; Roma-Rodrigues et al., 2019). Meanwhile, the hubs of the GO functional interaction networks (Figure 3B) showed that these genes, including Interleukin-6 (*IL-6*), BCL2 apoptosis regulator (*BCL2*), Interleukin-18 (*IL-18*), *CD33*, *CD44*, phosphodiesterase 4D (*PDE4D*) and Mitogen activated protein kinase 8 (*MAPK8*), participated in the immune and inflammation related signaling pathways.

### 5hmC as diagnostic biomarkers for DLBCL

**FIGURE 4:**
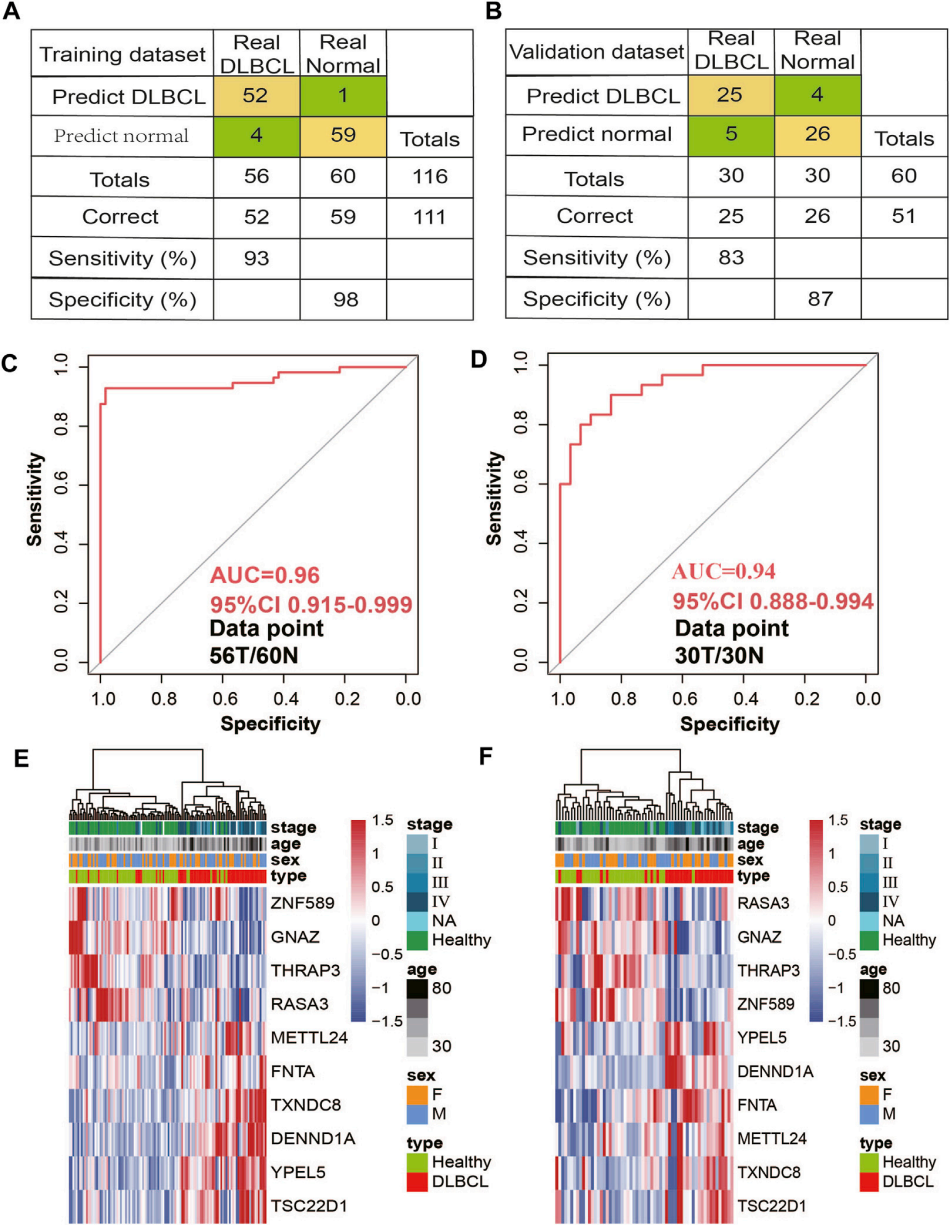
5hmC markers prediction for treatment response in the training and validation cohort. **(A,B)** Confusion tables of binary results of the diagnostic prediction model in the training **(A)** and validation data sets **(B)**. **(C,D)** Receiver operating characteristic (ROC) curve of the classifcation model with 10 5hmC markers in the training **(C)** and validation **(D)** cohorts. The true-positive rate (sensitivity) is plotted in function of the false-positive rate (1-specifcity). **(D)**. **(E,F)**, Unsupervised hierarchical clustering of 10 5hmC markers selected for use in the diagnostic prediction model in the training **(E)** and validation data sets **(F)**.

First, using RFECV based on logistic regression CV estimator, we reduced the number of DhMRs (Supplementary Table S2) in the training cohort, which achieved the best cross-validation score. Then, Using a logistic regression method, we constructed a diagnostic model with these ten markers. Applying the model yielded a sensitivity of 93% and specificity of 98% for DLBCL in the training data set of 56 DLBCL and 60 normal samples (Figure 4A) and a sensitivity of 83% and specificity of 87% in the validation data set of 30 DLBCL and 30 normal samples (Figure 4B). Subsequently, We also demonstrated this model could differentiate DLBCL patients from normal controls both in the training data set (AUC = 0.96) and the validation data set (AUC = 0.94) (Figures 4C,D). Unsupervised hierarchical clustering of these 10 markers was able to distinguish DLBCL from normal controls with high specificity and sensitivity. (Figures 4E,F). Finally, we also calculated the individual AUC for each of the 10 5hmC markers in the training and validation cohorts (Supplementary Figure S2B). Among these, THRAP3 showed the best diagnostic performance, yielding an AUC of 0.711 in the validation cohort (Supplementary Figure S2). These results indicate that plasma cfDNA-based 5hmC markers are a promising diagnostic tool for DLBCL. Meantime, we also applied this model to calculate the wd-score for every single sample, and showed that the wd-score in DLBCL patients was significantly higher than those in healthy volunteers (Supplementary Figure S3A). In addition, we also used the wd-score value to draw the box plot of different stages of the disease and clinical diagnostic indicators. The results showed that each stage and clinical diagnostic indicators were significantly different from the wd-score value of the healthy volunteers, but there was no difference between the groups (Supplementary Figure S3B).

### Potential associations between 5hmC markers and DLBCL

**FIGURE 5:**
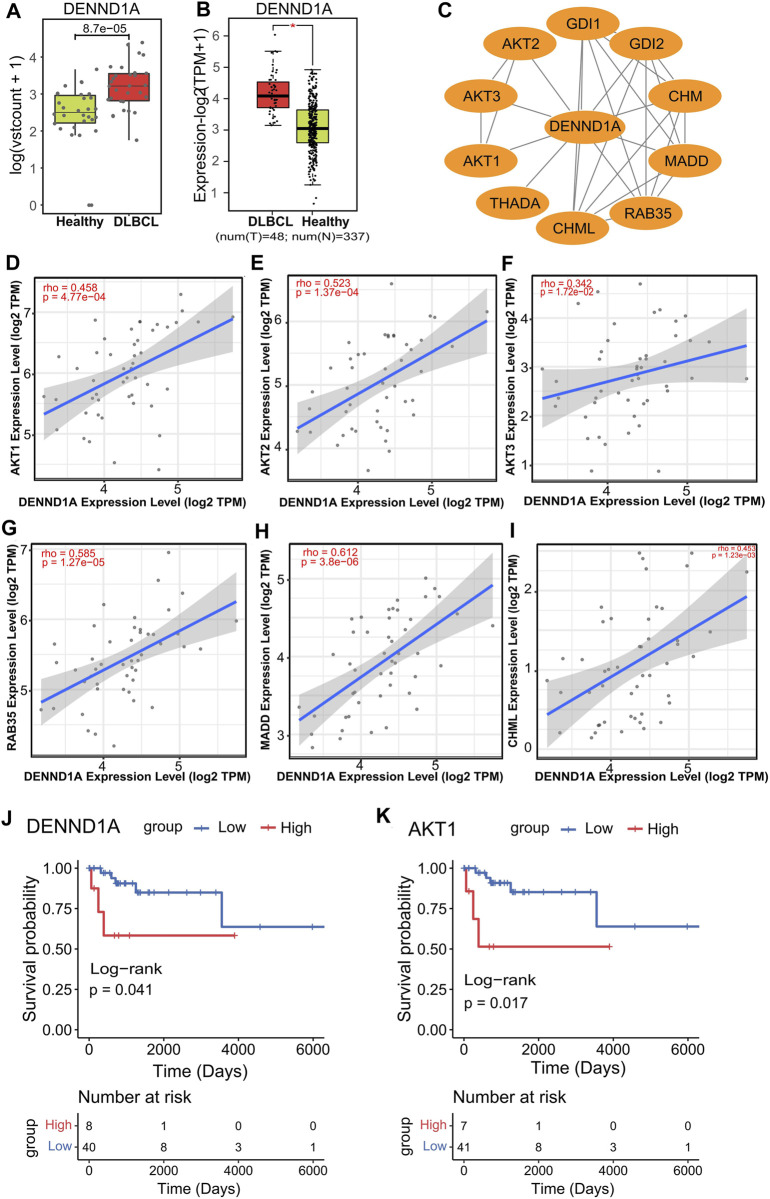
*DENND1A* and its potential relevance in DLBCL patients and treatment response. **(A,B)**, Box plot of *DENND1A*. Log2 transformed of TMM normalized 5hmC enrichment values were plotted, and Wilcoxon *t*-test was used (left). *DENND1A* mRNA expression level in the TCGA-DLBC dataset (right). **(C)**, Functional protein–protein interaction networks (PPI) from the STRING database. **(D–I)**, Correlation plots of the mRNA expression of *DENND1A* with the mRNA expressions of genes, including *AKT1, AKT2, AKT3, RAB35, MADD* and *CHML* in DLBCL in the TCGA-DLBC dataset. **(J,K)**, Overall survival curves of DLBCL patients with low or high gene expressions in *DENND1A* or *AKT1* in the TCGA-DLBC dataset.

Next, we sought to investigate the potential association of those 10 markers with DLBCL. Since our previous data showed that significantly expressed hydroxymethylation genes were mainly concentrated in immune-related signaling pathways, it was believed that these genes were related to DLBCL immune response. Therefore, we first intersected these 10 genes with immune-related genes to obtain two genes, *DENND1A* (DENN domain containing 1A) and *TSC22D1* (TSC22 domain family member 1) (Supplementary Figure S2A), and then among the two 5hmC-modified marker genes, *DENND1A* had the higher AUC in the validation cohort (Supplementary Figure S2B). In our study, *DENND1A* was highly enriched in hydroxymethylation in the DLBCL patients (*p* = 4.7e-08) (Figure 5A) and its mRNA expression level in the TCGA-DLBC dataset was consistent with the hydroxymethylation level in our data-set (Figure 5B).

In addition, from the PPI network constructed from the STRING database, we identifed several genes linked to *DENND1A*, including AKT serine/threonine kinase 1 (*AKT1*), AKT serine/threonine kinase 2 (*AKT2*), AKT serine/threonine kinase 3 (*AKT3*), Ras-related protein Rab-35 (*RAB35*), MAP kinase activating death domain (*MADD*) and CHM like Rab escort protein (CHML) (Figure 5C). Interestingly, we found that all of these gene expressions (*AKT1* (rho = 0.485), *AKT2* (rho = 0.523), *AKT3* (rho = 0.342), *RAB35* (rho = 0.585), *CHML* (rho = 0.453), *MADD* (rho = 0.612)) were positively associated with that of *DENND1A* (Figures 5D–I) (Supplementary Figure S4A). Moreover, from survival analysis results in the TCGA DLBC dataset, we found that the overall survival time (OS, days) of patients with high expression of *DENND1A* and *AKT1* was significantly lower than that of patients with low expression in these two genes (Figures 5J,K).

## Discussion

cfDNA in the circulating blood originates from dying cells from different tissues, which release DNA into the peripheral bloodstream upon degradation after cell death (Aucamp et al., 2018). Recent studies have reported that 5hmC in plasma cfDNA plays a critical role in gene expression regulation and is also a novel tool to identify biomarkers for disease diagnosis and prognosis (Han et al., 2016). In this study, we utilized a sensitive 5hmC-Seal method (Takahara et al., 2023) to generate the genome-wide profiles of cell-free 5hmC in DLBCL patients and healthy controls.

Our study proved that the 5hmC signals were enriched in the promoter, exons, UTR, and TTS regions. DLBCL patients and healthy controls showed significant differences in 5hmC enrichment, including 1,132 differentially hydroxymethylated genes (DhMGs) detected by differential analysis method. Additionally, GO analysis of those 1,132 marker genes with differentially modified 5hmC between DLBCL patients and healthy controls suggested enrichment in immune and inflammation-related signaling pathways, such as myeloid leukocyte activation, CD4-positive alpha-beta T cell activation, peptidyl-serine modification. There is a broad consensus in cancer research that inflammation and immune response facilitate tumor progression, infiltration, and metastasis via different mechanisms (Bi et al., 2022), and tumor progression is also highly correlated with the physiological state of tumor microenvironment (TME) (Xiao and Yu, 2021). The TME is composed of complex components, mainly including tumor cells, interstitial factors, extracellular matrix, inflammatory and immune cells, etc., (Cioroianu et al., 2019). Some studies have reported that immune cell subtypes and immune-related signaling pathways in the tumor microenvironment of DLBCL are related to its progression and prognosis (Uddin et al., 2006; Hasselblom et al., 2010). Importantly, cfDNA is not only derived from tumor cells, but also from the tumor microenvironment (Gahan and Swaminathan, 2008). Therefore, these 5hmC modified genes could be related to the progression of DLBCL.

Furthermore, we found that ten 5hmC markers filtered by machine learning algorithms could well distinguish DLBCL patients from healthy controls in both the training and validation cohorts. Meantime, the prediction performance of the logistic regression model, established by 10 5hmC markers, achieving 83% sensitivity and 87% specificity (AUC = 0.94). Due to in the clinic, digital evaluation criteria would be more preferred, so a wd-score was then computed according to the logistic model coefficients and modification level of the corresponding markers for each individual. We speculated that 5hmC markers identified in this study might be used for the early diagnosis of DLBCL given that a significant difference in wd-scores between DLBCL patients and healthy volunteers was observed. Taken together, these findings indicated that 5hmC markers derived from cfDNA may serve as effective epigenetic biomarkers for minimally noninvasive diagnosis for DLBCL.

According to recent studies, the regulation of immune response and function plays a key role in the pathogenesis and progression of DLBCL (Kulasekaran et al., 2015; Cai et al., 2019; Zeng et al., 2019), and our data are also concentrated in immune-related functional regions. Therefore, in this study, we intersected the above 10 biomarkers with immune-related genes in the database, and found that *TSC22D1* and *DENND1A* were associated with immunity. Notably, among this two 5hmC modified genes, *DENND1A* showed the best predictive performance. Meantime, *DENND1A* expression was positively correlated with *AKT1, AKT2, AKT3, RAB35,* and *CHML*. Recent work suggested constitutive activation of the PI3K/protein kinase B (*AKT*) pathway that plays a crucial role in mediating growth, proliferation, and cell survival in a substantial number of DLBCL patient samples (Langmead and Salzberg, 2012; Bi et al., 2022). Interestingly, Functional study of *DENND1A* found that *DENND1A* acted as a guanine nucleotide exchange factor for *RAB35*, which activated *RAB35* and regulated the activation of *AKT*, a key protein in PI3K signaling pathway. After down-regulating the expression of *RAB35*, *AKT* activity was decreased (Steen et al., 2021; Xiao and Yu, 2021). Other studies have also found that *RAB35* is a new regulator of PI3K pathway, the depletion of *RAB35* can inhibit *AKT* phosphorylation, while the expression of *RAB35* mutants activates the PI3K/AKT pathway, and *RAB35* plays a role in the downstream of growth factor receptors and the upstream of *PDK1*. *RAB35* was co-expressed with PI3K in immunoprecipitation assay (Steen et al., 2021). Taken together, these data indicate that *DENND1A* as *RAB35* guanine nucleotide exchange factor, may participate in regulating the PI3K/AKT signaling pathway affecting DLBCL progress, but its specific mechanism is unclear.

Nevertheless, there are limitations to our study. Firstly, the number of DLBCL patients is relatively small and may not fully represent all DLBCL patients. Recently, 5hmC has become a novel class of cancer epigenetic biomarkers. Compared with the clinical gold standard, it has high sensitivity and specificity in the early detection of some cancers (Scholler et al., 2022), and has shown the potential for the diagnosis and prognosis of different diseases (Gahan and Swaminathan, 2008), which has application prospects in the field of precision medicine. Thus, the performance of our model still needs to be tested in larger study cohorts. Secondly, In this study, we included a small number of patients in each stage, which may have a certain impact on the current diagnostic results of DLBCL. In addition, considering that the early diagnosis of DLBCL is of great significance, we will include more samples of early DLBCL patients in the later study, in order to achieve the early diagnosis of DLBCL by minimally invasive means. Thirdly, this study only focuses on Chinese patients and may not represent DLBCL patients in other races, and therefore more validation will be necessary to demonstrate the generalizability of the results in prospective studies which will cover other populations, geographical regions, and disease risk factors. Fourthly, In the clinical cohort of this study, we have not included samples of other types of blood tumors. In future studies, we plan to broaden the sample range to encompass early-stage diffuse large B-cell lymphoma (DLBCL) as well as other blood tumors in order to validate and establish the reliability of the 5hmC marker for early diagnosis of DLBCL patients.Finally, the regulatory mechanism of 5hmC in *DENND1A* is still not clear. In the future, we aim to increase the sample size of DLBCL patients and find more stable and reliable 5hmC marker genes to diagnose DLBCL patients.

## Conclusion

In conclusions, our research indicated that 5hmC signatures in plasma cfDNA can be served as effective biomarkers for non-invasive diagnosis of DLBCL. Our findings have the potential to be the development of new strategies for diagnosis and therapeutic treatment of DLBCL.

## Data Availability

The raw sequence data reported in this paper have been deposited in the Genome Sequence Archive of the BIG Data Center at the Beijing Institute of Genomics, Chinese Academy of Science, under accession number HRA007682 (accessible at https://ngdc.cncb.ac.cn/gsa-human). Code is available from the corresponding author on reason able request.

## Supplementary material

SUPPLEMENTARY FIGURE S1 Genomic distribution of 5-hmC in DLBCL patients and healthy controls. **(A–D)**, Venn diagram showing gene number associated with peaks measured for those four groups; peaks that overlap with 3′-UTR, 5′-UTR, exon, and promoter were considered for the analysis.

SUPPLEMENTARY FIGURE S2 The individual prediction performance of each marker in the thirteen markers in the training and validation cohort. **(A)**, The venn diagram in the upper left corner shows the intersection of immune-related genes and 10 5hmC markers. This intersection yielded two markers, *DENND1A* and *TSC22D1*. **(B–K)**, The other graphs represent the Individual ROC curves for each marker in the training and validation cohort.

SUPPLEMENTARY FIGURE S3 The wd-scores information. **(A)**, Boxplot of wd-score deriving from the integrated model for the DLBCL samples and the healthy controls. **(B–E)**, box plot of different stages of the disease and clinical diagnostic indicators. GCB: Germinal Center B-cell-like, Non-GCB: Non-Germinal Center B-cell-like.

SUPPLEMENTARY FIGURE S4 Correlation plots of the mRNA expression of *DENND1A* with the mRNA expressions of *GDI1, GDI2, THADA* and *CHM* in DLBCL in the TCGA-DLBC dataset. **(A)**, *GDI1*. **(B)**, *GDI2*. **(C)**, *THADA*. **(D)**, *CHM*.

## Abbreviations

cfDNA, Cell-free DNA; 5hmC, 5-Hydroxymethylcytosine; 5mC, 5-Methylcytosine; AUC, Area under ROC curves; DLBCL, Diffuse Large B-Cell Lymphoma; STRING, Search Tool for the Retrieval of Interacting Genes; LDH, Lactate dehydrogenase; β2MG, β2 Microglobulin; IPI, International Prognostic Index; DhMRs, Deferentially 5hMc-enriched regions; DhMGs, Differentially hydroxymethylated genes; OS, Overall survival.

## References

[B1] Abraham A. Pedregosa F. Eickenberg M. Gervais P. Mueller A. Kossaifi J. (2014). Machine learning for neuroimaging with scikit-learn. Front. Neuroinform 8, 14. 10.3389/fninf.2014.00014 24600388 PMC3930868

[B2] An Y. Zhao X. Zhang Z. Xia Z. Yang M. Ma L. (2023). DNA methylation analysis explores the molecular basis of plasma cell-free DNA fragmentation. Nat. Commun. 14 (1), 287. 10.1038/s41467-023-35959-6 36653380 PMC9849216

[B3] Aucamp J. Bronkhorst A. J. Badenhorst C. P. S. Pretorius P. J. (2018). The diverse origins of circulating cell-free DNA in the human body: a critical re-evaluation of the literature. Biol. Rev. Camb Philos. Soc. 93 (3), 1649–1683. 10.1111/brv.12413 29654714

[B4] Barakat A. Mittal A. Ricketts D. Rogers B. A. (2019). Understanding survival analysis: actuarial life tables and the Kaplan-Meier plot. Br. J. Hosp. Med. (Lond) 80 (11), 642–646. 10.12968/hmed.2019.80.11.642 31707885

[B5] Bi Q. Wu J. Y. Qiu X. M. Zhang J. D. Sun Z. J. Wang W. (2022). Tumor-associated inflammation: the tumor-promoting immunity in the early stages of tumorigenesis. J. Immunol. Res. 2022, 3128933. 10.1155/2022/3128933 35733919 PMC9208911

[B6] Bohers E. Viailly P. J. Dubois S. Bertrand P. Maingonnat C. Mareschal S. (2015). Somatic mutations of cell-free circulating DNA detected by next-generation sequencing reflect the genetic changes in both germinal center B-cell-like and activated B-cell-like diffuse large B-cell lymphomas at the time of diagnosis. Haematologica 100 (7), e280–e284. 10.3324/haematol.2015.123612 25749829 PMC4486242

[B7] Cai J. Chen L. Zhang Z. Zhang X. Lu X. Liu W. (2019). Genome-wide mapping of 5-hydroxymethylcytosines in circulating cell-free DNA as a non-invasive approach for early detection of hepatocellular carcinoma. Gut 68 (12), 2195–2205. 10.1136/gutjnl-2019-318882 31358576 PMC6872444

[B8] Chen H. Y. Zhang W. L. Zhang L. Yang P. Li F. Yang Z. R. (2021). 5-Hydroxymethylcytosine profiles of cfDNA are highly predictive of R-CHOP treatment response in diffuse large B cell lymphoma patients. Clin. Epigenetics 13 (1), 33. 10.1186/s13148-020-00973-8 33573703 PMC7879534

[B9] Chen K. Zhang J. Guo Z. Ma Q. Xu Z. Zhou Y. (2016). Loss of 5-hydroxymethylcytosine is linked to gene body hypermethylation in kidney cancer. Cell Res. 26 (1), 103–118. 10.1038/cr.2015.150 26680004 PMC4816137

[B10] Chiu B. C. Zhang Z. You Q. Zeng C. Stepniak E. Bracci P. M. (2019). Prognostic implications of 5-hydroxymethylcytosines from circulating cell-free DNA in diffuse large B-cell lymphoma. Blood Adv. 3 (19), 2790–2799. 10.1182/bloodadvances.2019000175 31570490 PMC6784517

[B11] Cioroianu A. I. Stinga P. I. Sticlaru L. Cioplea M. D. Nichita L. Popp C. (2019). Tumor microenvironment in diffuse large B-cell lymphoma: role and prognosis. Anal. Cell Pathol. (Amst) 2019, 8586354. 10.1155/2019/8586354 31934533 PMC6942707

[B12] Cristiano S. Leal A. Phallen J. Fiksel J. Adleff V. Bruhm D. C. (2019). Genome-wide cell-free DNA fragmentation in patients with cancer. Nature 570 (7761), 385–389. 10.1038/s41586-019-1272-6 31142840 PMC6774252

[B13] Diaz L. A. Jr. Bardelli A. (2014). Liquid biopsies: genotyping circulating tumor DNA. J. Clin. Oncol. 32 (6), 579–586. 10.1200/jco.2012.45.2011 24449238 PMC4820760

[B14] Fang C. Xu W. Li J. Y. (2010). A systematic review and meta-analysis of rituximab-based immunochemotherapy for subtypes of diffuse large B cell lymphoma. Ann. Hematol. 89 (11), 1107–1113. 10.1007/s00277-010-0990-5 20499236

[B15] Gahan P. B. Swaminathan R. (2008). Circulating nucleic acids in plasma and serum. Recent developments. Ann. N. Y. Acad. Sci. 1137, 1–6. 10.1196/annals.1448.050 18837917

[B16] Goldman M. J. Craft B. Hastie M. Repečka K. McDade F. Kamath A. (2020). Visualizing and interpreting cancer genomics data via the Xena platform. Nat. Biotechnol. 38 (6), 675–678. 10.1038/s41587-020-0546-8 32444850 PMC7386072

[B17] Han D. Lu X. Shih A. H. Nie J. You Q. Xu M. M. (2016). A highly sensitive and robust method for genome-wide 5hmC profiling of rare cell populations. Mol. Cell 63 (4), 711–719. 10.1016/j.molcel.2016.06.028 27477909 PMC4992443

[B18] Hasselblom S. Hansson U. Olsson M. Torén L. Bergström A. Nilsson-Ehle H. (2010). High immunohistochemical expression of p-AKT predicts inferior survival in patients with diffuse large B-cell lymphoma treated with immunochemotherapy. Br. J. Haematol. 149 (4), 560–568. 10.1111/j.1365-2141.2010.08123.x 20201946

[B19] Kristensen L. S. Hansen J. W. Kristensen S. S. Tholstrup D. Harsløf L. B. Pedersen O. B. (2016). Aberrant methylation of cell-free circulating DNA in plasma predicts poor outcome in diffuse large B cell lymphoma. Clin. Epigenetics 8 (1), 95. 10.1186/s13148-016-0261-y 27610206 PMC5015248

[B20] Kulasekaran G. Nossova N. Marat A. L. Lund I. Cremer C. Ioannou M. S. (2015). Phosphorylation-dependent regulation of connecdenn/DENND1 guanine nucleotide exchange factors. J. Biol. Chem. 290 (29), 17999–18008. 10.1074/jbc.M115.636712 26055712 PMC4505046

[B21] Langmead B. (2010). Aligning short sequencing reads with Bowtie. Curr. Protoc. Bioinforma. Chapter 11, Unit 11.7. 10.1002/0471250953.bi1107s32 PMC3010897 21154709

[B22] Langmead B. Salzberg S. L. (2012). Fast gapped-read alignment with Bowtie 2. Nat. Methods 9 (4), 357–359. 10.1038/nmeth.1923 22388286 PMC3322381

[B23] Li H. Handsaker B. Wysoker A. Fennell T. Ruan J. Homer N. (2009). The sequence alignment/map format and SAMtools. Bioinformatics 25 (16), 2078–2079. 10.1093/bioinformatics/btp352 19505943 PMC2723002

[B24] Li T. Fan J. Wang B. Traugh N. Chen Q. Liu J. S. (2017). TIMER: a web server for comprehensive analysis of tumor-infiltrating immune cells. Cancer Res. 77 (21), e108–e110. 10.1158/0008-5472.Can-17-0307 29092952 PMC6042652

[B25] Li W. Zhang X. Lu X. You L. Song Y. Luo Z. (2017). 5-Hydroxymethylcytosine signatures in circulating cell-free DNA as diagnostic biomarkers for human cancers. Cell Res. 27 (10), 1243–1257. 10.1038/cr.2017.121 28925386 PMC5630683

[B26] Li W. Zhang X. Lu X. You L. Song Y. Luo Z. (2019). Author Correction: 5-Hydroxymethylcytosine signatures in circulating cell-free DNA as diagnostic biomarkers for human cancers. Cell Res. 29 (7), 599. 10.1038/s41422-019-0182-3 31110249 PMC6796932

[B27] Lo Y. M. D. Han D. S. C. Jiang P. Chiu R. W. K. (2021). Epigenetics, fragmentomics, and topology of cell-free DNA in liquid biopsies. Science 372 (6538), eaaw3616. 10.1126/science.aaw3616 33833097

[B28] Loyfer N. Magenheim J. Peretz A. Cann G. Bredno J. Klochendler A. (2023). A DNA methylation atlas of normal human cell types. Nature 613 (7943), 355–364. 10.1038/s41586-022-05580-6 36599988 PMC9811898

[B29] Luo H. Wei W. Ye Z. Zheng J. Xu R. H. (2021). Liquid biopsy of methylation biomarkers in cell-free DNA. Trends Mol. Med. 27 (5), 482–500. 10.1016/j.molmed.2020.12.011 33500194

[B30] Malpica L. Marques-Piubelli M. L. Beltran B. E. Chavez J. C. Miranda R. N. Castillo J. J. (2022). EBV-positive diffuse large B-cell lymphoma, not otherwise specified: 2022 update on diagnosis, risk-stratification, and management. Am. J. Hematol. 97 (7), 951–965. 10.1002/ajh.26579 35472248

[B31] Quail D. F. Joyce J. A. (2013). Microenvironmental regulation of tumor progression and metastasis. Nat. Med. 19 (11), 1423–1437. 10.1038/nm.3394 24202395 PMC3954707

[B32] Roma-Rodrigues C. Mendes R. Baptista P. V. Fernandes A. R. (2019). Targeting tumor microenvironment for cancer therapy. Int. J. Mol. Sci. 20 (4), 840. 10.3390/ijms20040840 30781344 PMC6413095

[B33] Schmitz R. Wright G. W. Huang D. W. Johnson C. A. Phelan J. D. Wang J. Q. (2018). Genetics and pathogenesis of diffuse large B-cell lymphoma. N. Engl. J. Med. 378 (15), 1396–1407. 10.1056/NEJMoa1801445 29641966 PMC6010183

[B34] Scholler N. Perbost R. Locke F. L. Jain M. D. Turcan S. Danan C. (2022). Tumor immune contexture is a determinant of anti-CD19 CAR T cell efficacy in large B cell lymphoma. Nat. Med. 28 (9), 1872–1882. 10.1038/s41591-022-01916-x 36038629 PMC9499856

[B35] Sehn L. Salles G. (2021). Diffuse large B-cell lymphoma. N. Engl. J. Med. 384 (9), 842–858. 10.1056/NEJMra2027612 33657296 PMC8377611

[B36] Snyder M. W. Kircher M. Hill A. J. Daza R. M. Shendure J. (2016). Cell-free DNA comprises an *in vivo* nucleosome footprint that informs its tissues-of-origin. Cell 164 (1-2), 57–68. 10.1016/j.cell.2015.11.050 26771485 PMC4715266

[B37] Song C. X. Szulwach K. E. Fu Y. Dai Q. Yi C. Li X. (2011). Selective chemical labeling reveals the genome-wide distribution of 5-hydroxymethylcytosine. Nat. Biotechnol. 29 (1), 68–72. 10.1038/nbt.1732 21151123 PMC3107705

[B38] Song C. X. Yin S. Ma L. Wheeler A. Chen Y. Zhang Y. (2017). 5-Hydroxymethylcytosine signatures in cell-free DNA provide information about tumor types and stages. Cell Res. 27 (10), 1231–1242. 10.1038/cr.2017.106 28820176 PMC5630676

[B39] Steen C. B. Luca B. A. Esfahani M. S. Azizi A. Sworder B. J. Nabet B. Y. (2021). The landscape of tumor cell states and ecosystems in diffuse large B cell lymphoma. Cancer Cell 39 (10), 1422–1437.e10. 10.1016/j.ccell.2021.08.011 34597589 PMC9205168

[B40] Sun K. Jiang P. Chan K. C. Wong J. Cheng Y. K. Liang R. H. (2015). Plasma DNA tissue mapping by genome-wide methylation sequencing for noninvasive prenatal, cancer, and transplantation assessments. Proc. Natl. Acad. Sci. U. S. A. 112 (40), E5503–E5512. 10.1073/pnas.1508736112 26392541 PMC4603482

[B41] Takahara T. Nakamura S. Tsuzuki T. Satou A. (2023). The immunology of DLBCL. Cancers (Basel) 15 (3), 835. 10.3390/cancers15030835 36765793 PMC9913124

[B42] Torbicki A. Bacchi M. Delcroix M. Farber H. W. Ghofrani H. A. Hennessy B. (2019). Integrating data from randomized controlled trials and observational studies to assess survival in rare diseases. Circ. Cardiovasc Qual. Outcomes 12 (5), e005095. 10.1161/circoutcomes.118.005095 31109190 PMC7665498

[B43] Tuchalska-Czuroń J. Lenart J. Augustyniak J. Durlik M. (2020). Clinical value of tissue DNA integrity index in pancreatic cancer. Surgeon 18 (5), 269–279. 10.1016/j.surge.2019.10.008 32156475

[B44] Uddin S. Hussain A. R. Siraj A. K. Manogaran P. S. Al-Jomah N. A. Moorji A. (2006). Role of phosphatidylinositol 3’-kinase/AKT pathway in diffuse large B-cell lymphoma survival. Blood 108 (13), 4178–4186. 10.1182/blood-2006-04-016907 16946303

[B45] Vasanthakumar A. Godley L. A. (2015). 5-hydroxymethylcytosine in cancer: significance in diagnosis and therapy. Cancer Genet. 208 (5), 167–177. 10.1016/j.cancergen.2015.02.009 25892122

[B46] Wedge E. Hansen J. W. Garde C. Asmar F. Tholstrup D. Kristensen S. S. (2017). Global hypomethylation is an independent prognostic factor in diffuse large B cell lymphoma. Am. J. Hematol. 92 (7), 689–694. 10.1002/ajh.24751 28378885

[B47] Xiao Y. Yu D. (2021). Tumor microenvironment as a therapeutic target in cancer. Pharmacol. Ther. 221, 107753. 10.1016/j.pharmthera.2020.107753 33259885 PMC8084948

[B48] Yeh P. Hunter T. Sinha D. Ftouni S. Wallach E. Jiang D. (2017). Circulating tumour DNA reflects treatment response and clonal evolution in chronic lymphocytic leukaemia. Nat. Commun. 8, 14756. 10.1038/ncomms14756 28303898 PMC5357854

[B49] Zemmour H. Planer D. Magenheim J. Moss J. Neiman D. Gilon D. (2018). Non-invasive detection of human cardiomyocyte death using methylation patterns of circulating DNA. Nat. Commun. 9 (1), 1443. 10.1038/s41467-018-03961-y 29691397 PMC5915384

[B50] Zeng C. Stroup E. K. Zhang Z. Chiu B. C. Zhang W. (2019). Towards precision medicine: advances in 5-hydroxymethylcytosine cancer biomarker discovery in liquid biopsy. Cancer Commun. (Lond) 39 (1), 12. 10.1186/s40880-019-0356-x 30922396 PMC6440138

[B51] Zhang Y. Liu T. Meyer C. A. Eeckhoute J. Johnson D. S. Bernstein B. E. (2008). Model-based analysis of ChIP-seq (MACS). Genome Biol. 9 (9), R137. 10.1186/gb-2008-9-9-r137 18798982 PMC2592715

[B52] Zhou Q. Kang G. Jiang P. Qiao R. Lam W. K. J. Yu S. C. Y. (2022). Epigenetic analysis of cell-free DNA by fragmentomic profiling. Proc. Natl. Acad. Sci. U. S. A. 119 (44), e2209852119. 10.1073/pnas.2209852119 36288287 PMC9636966

