# Supplementary figures and images for: 5-Hydroxymethylcytosine profilings in circulating cell-free DNA as diagnostic biomarkers for DLBCL
Source: Front Cell Dev Biol. 2024 Nov 15;12:1387959. doi: 10.3389/fcell.2024.1387959 (PMC11604450; doi:10.3389/fcell.2024.1387959)

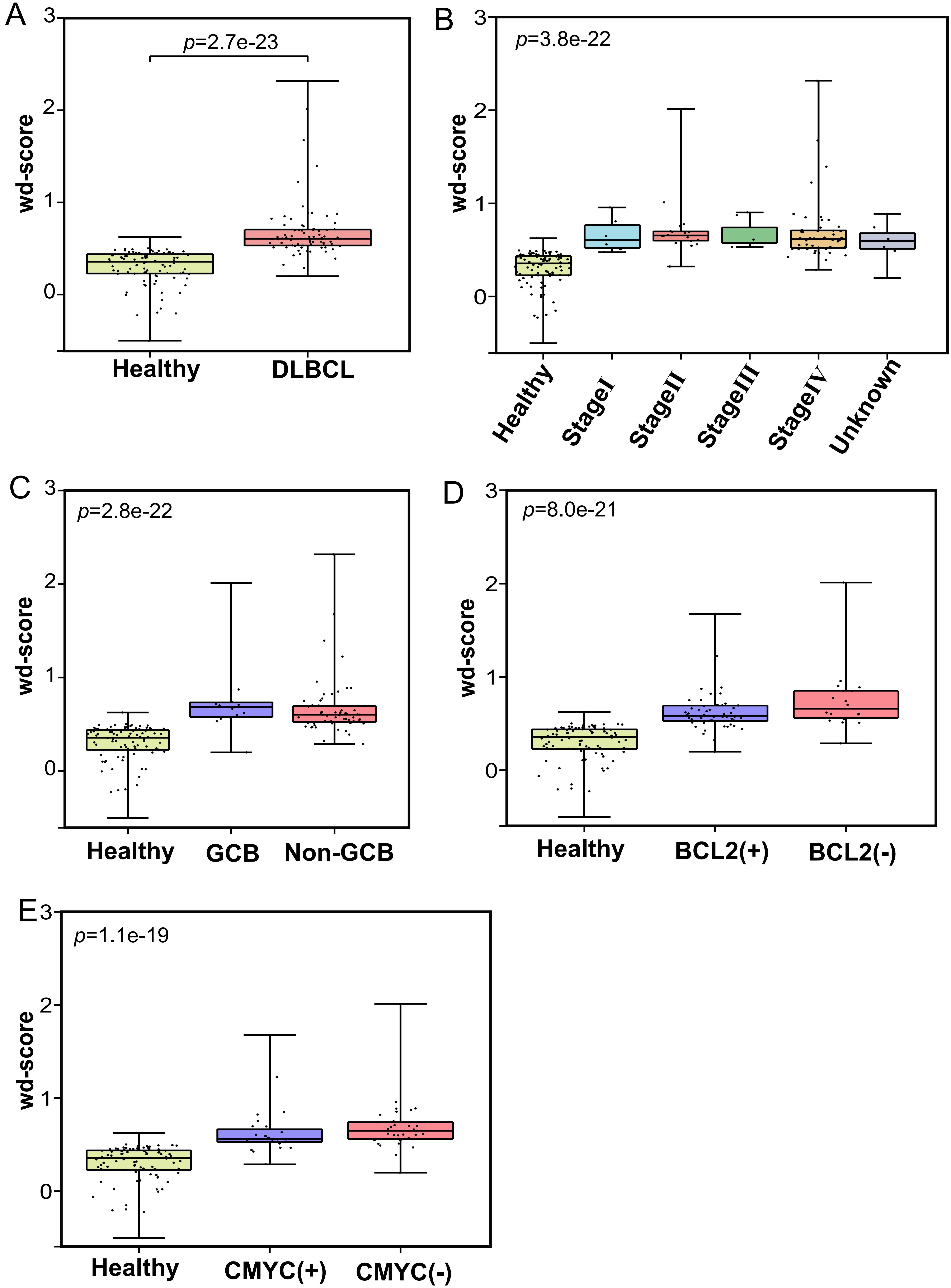

Supplement: Supplementary file 2 [file Image3.JPEG]

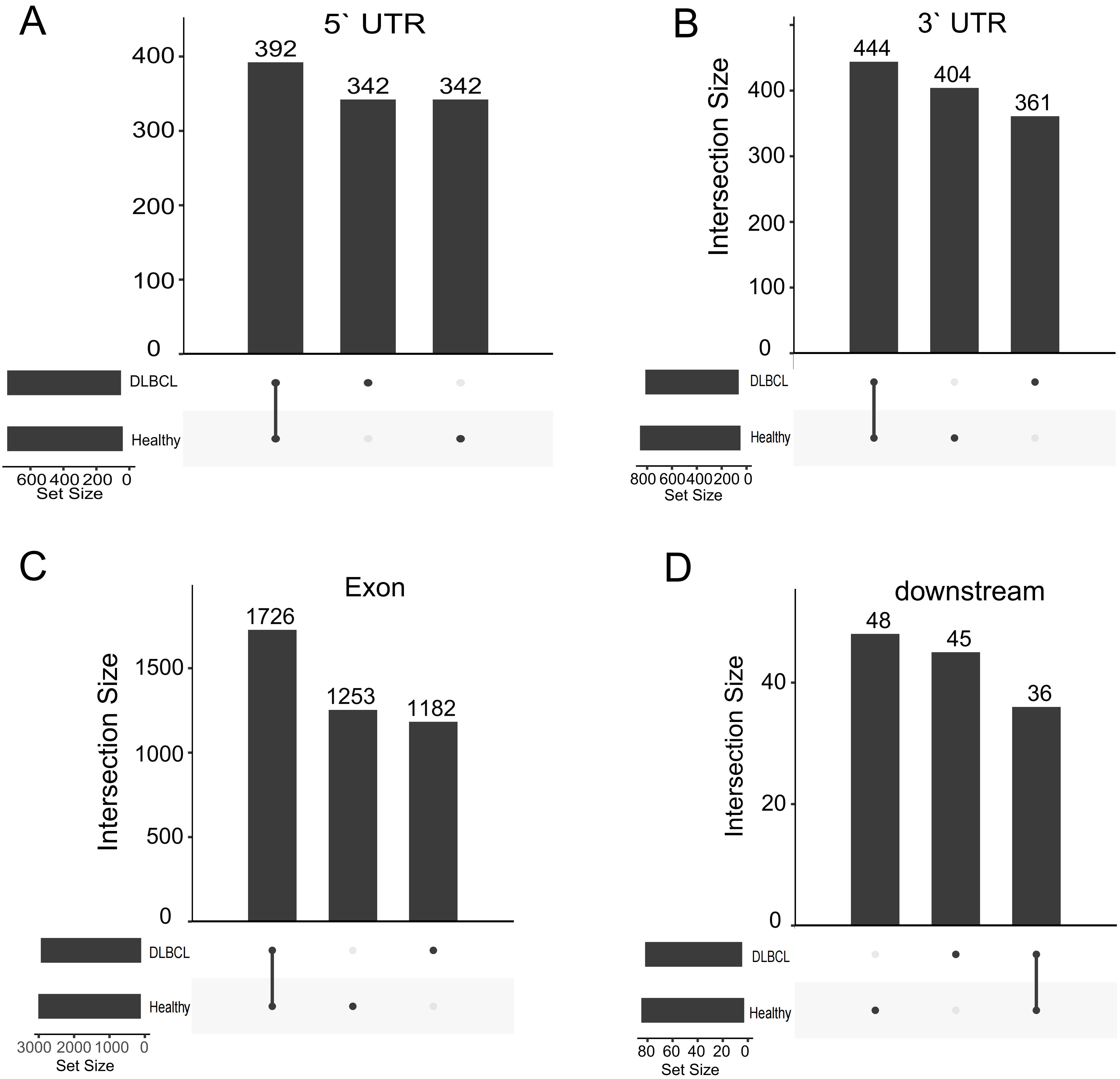

Supplement: Supplementary file 3 [file Image1.JPEG]

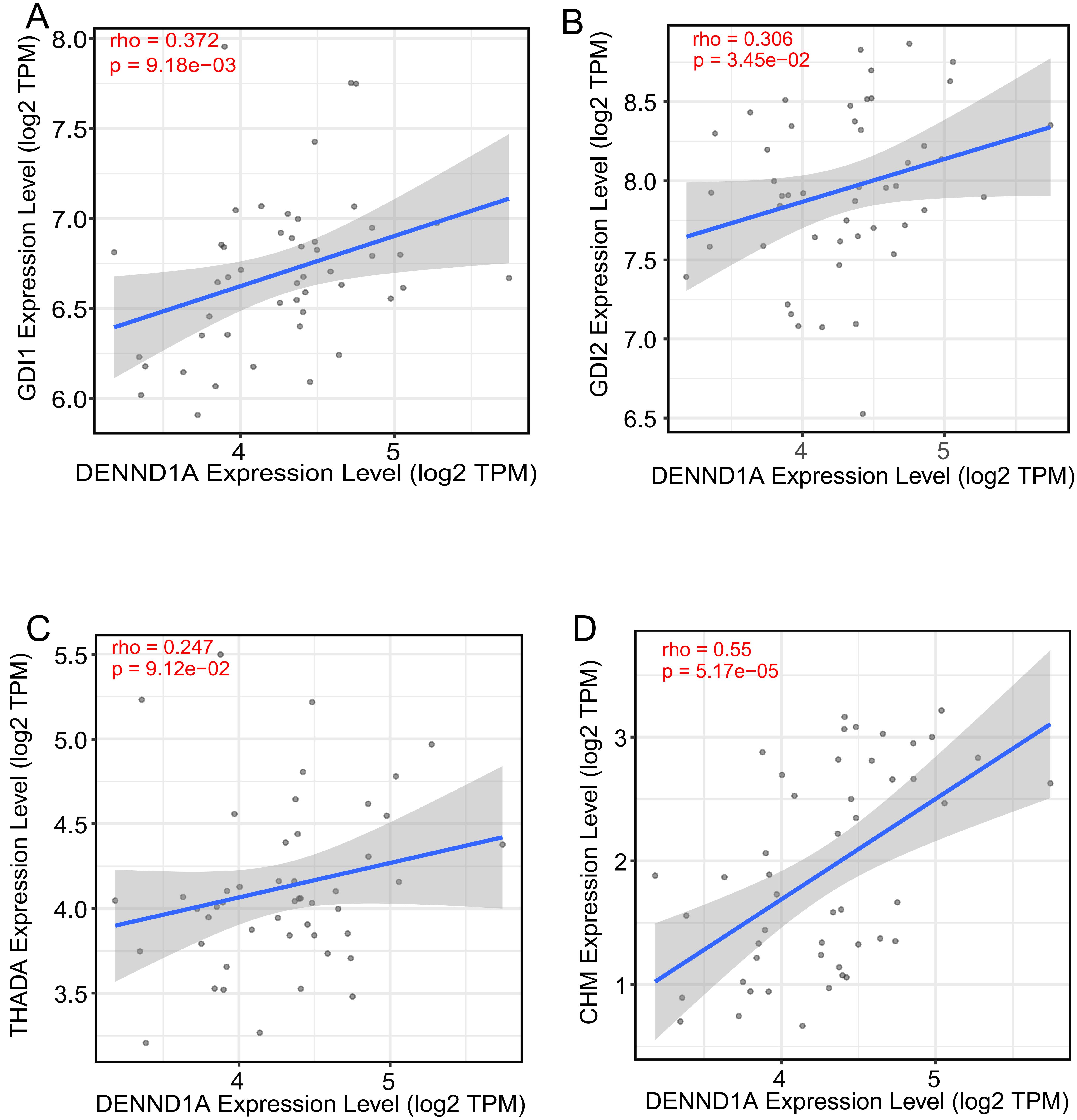

Supplement: Supplementary file 4 [file Image4.JPEG]

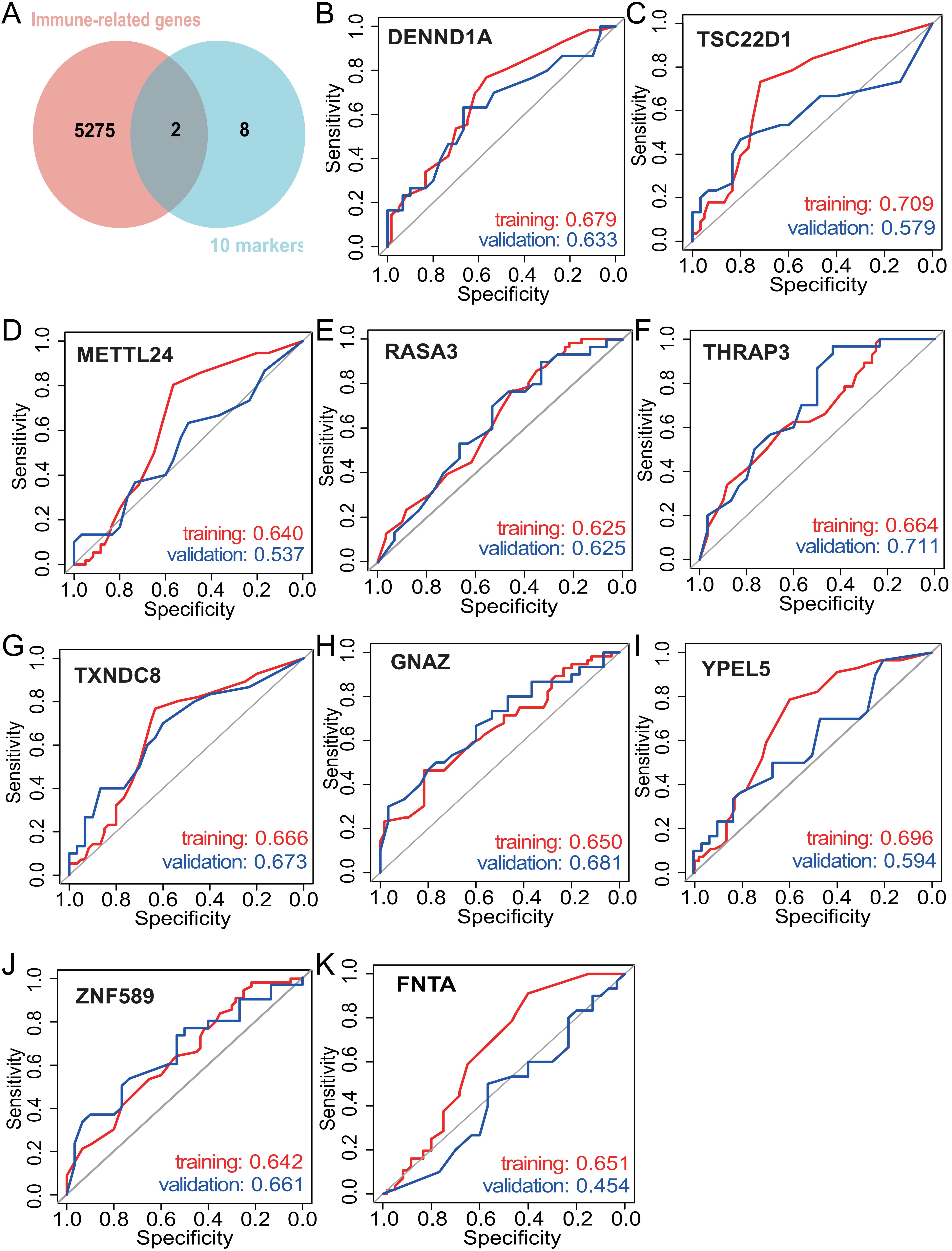

Supplement: Supplementary file 5 [file Image2.JPEG]
