# Supplementary material for: 5-Hydroxymethylcytosine profilings in circulating cell-free DNA as diagnostic biomarkers for DLBCL
Source: Front Cell Dev Biol. 2024 Nov 15;12:1387959. doi: 10.3389/fcell.2024.1387959 (PMC11604450; doi:10.3389/fcell.2024.1387959)
Supplement: Supplementary file 6 [file Table3.DOCX]

**5-hydroxymethylcytosine Profilings in Circulating Cell-free DNA as diagnostic biomarkers for DLBCL**

Hangyu Chen^1,2#^, Yangming Ding^2#^, Lei Zhang^2^, Xuehui Li^6^, Hong Xiao^5^, Subinuer Kuerban^6^, Baixin Zhen^6^, Yuxi Wang^2^, Long Chen^2^, Jian Lin^2,3,4,5^, Maimaitiyasen Duolikun^2,5*^, Hai-chuan Zhu^1*^

1 Institute of Biology and Medicine, College of Life and Health Sciences, Wuhan University of Science and Technology, Wuhan, China.

2 Department of Pharmacy, Peking University Third Hospital, Beijing, China.

3 Synthetic and Functional Biomolecules Center, Beijing National Laboratory for Molecular Sciences, Peking University, Beijing, China.

4 Peking University Third Hospital Cancer Center, Beijing, China.

5 Key Laboratory of Tropical Biological Resources of Ministry of Education, School of Pharmaceutical Sciences, Hainan University, Haikou, China.

6 College of Pharmacy，Xinjiang Medical University, Urumqi, China.

**# icon represents the first author**

*** Corresponding authors:**

**Hai-chuan Zhu, Ph.D., Email: [zhuhaichuan@wust.edu.cn](mailto:zhuhaichuan@wust.edu.cn)**

Institute of Biology and Medicine, College of Life and Health Sciences, Wuhan University of Science and Technology.

Add: 947 Heping Avenue, Qingshan District, Wuhan, 430081, China

**Maimaitiyasen Duolikun, B.Sc., Email: yasen@hainanu.edu.cn**

Department of Pharmacy, Peking University Third Hospital,

Key Laboratory of Tropical Biological Resources of Ministry of Education, School of Pharmaceutical Sciences, Hainan University.

Add: 49 Huayuan North Rd, Haidian District, Beijing 100191, China


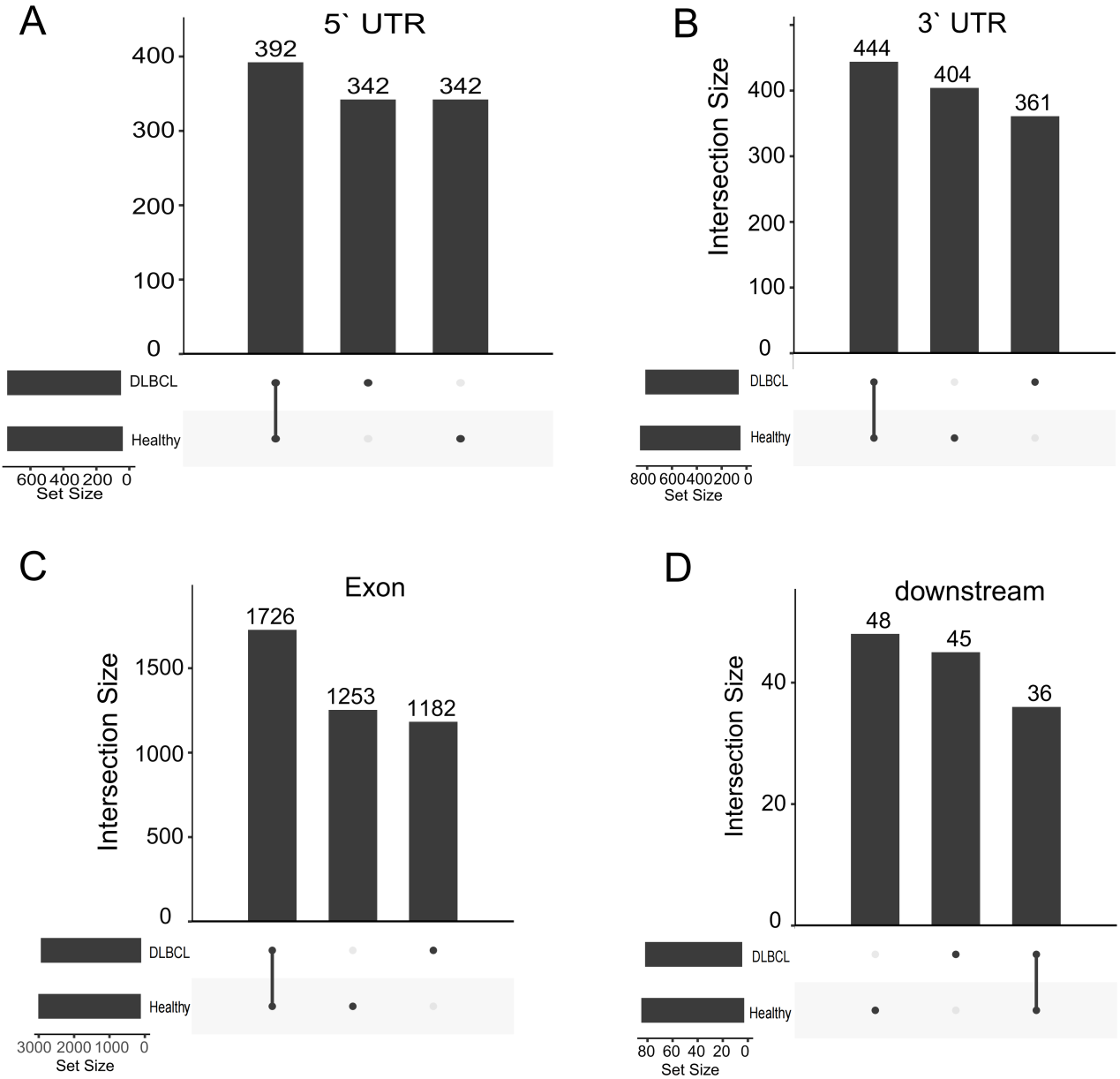


**Supplementary Figure 1. Genomic distribution of 5-hmC in DLBCL patients and healthy controls. A-D,** Venn diagram showing gene number associated with peaks measured for those four groups; peaks that overlap with 3′-UTR, 5′-UTR, exon, and promoter were considered for the analysis.


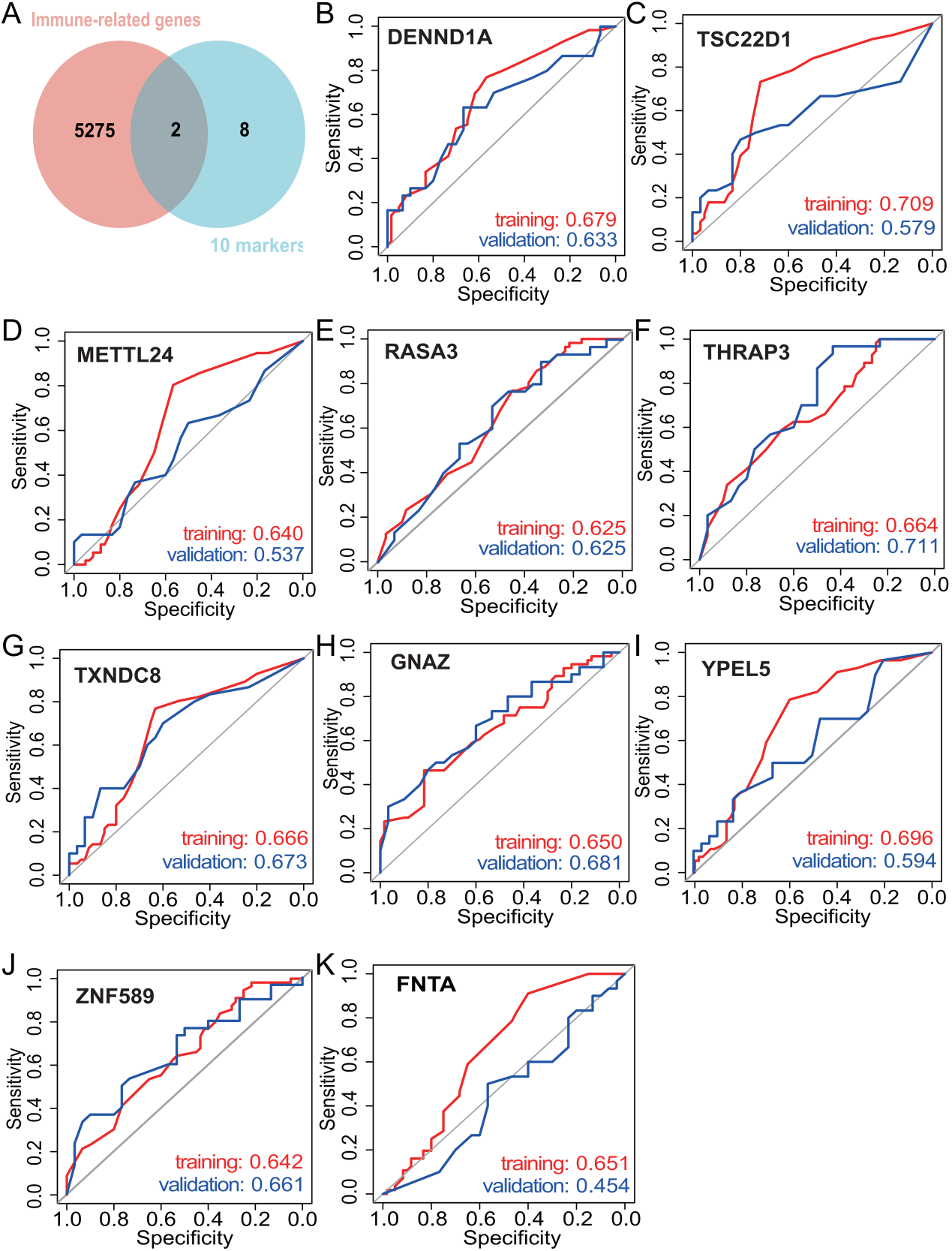


**Supplementary Figure 2. The individual prediction performance of each marker in the thirteen markers in the training and validation cohort. A,** The venn diagram in the upper left corner shows the intersection of immune-related genes and 10 5hmC markers. This intersection yielded two markers, *DENND1A* and *TSC22D1*. **B-K,** The other graphs represent the Individual ROC curves for each marker in the training and validation cohort.


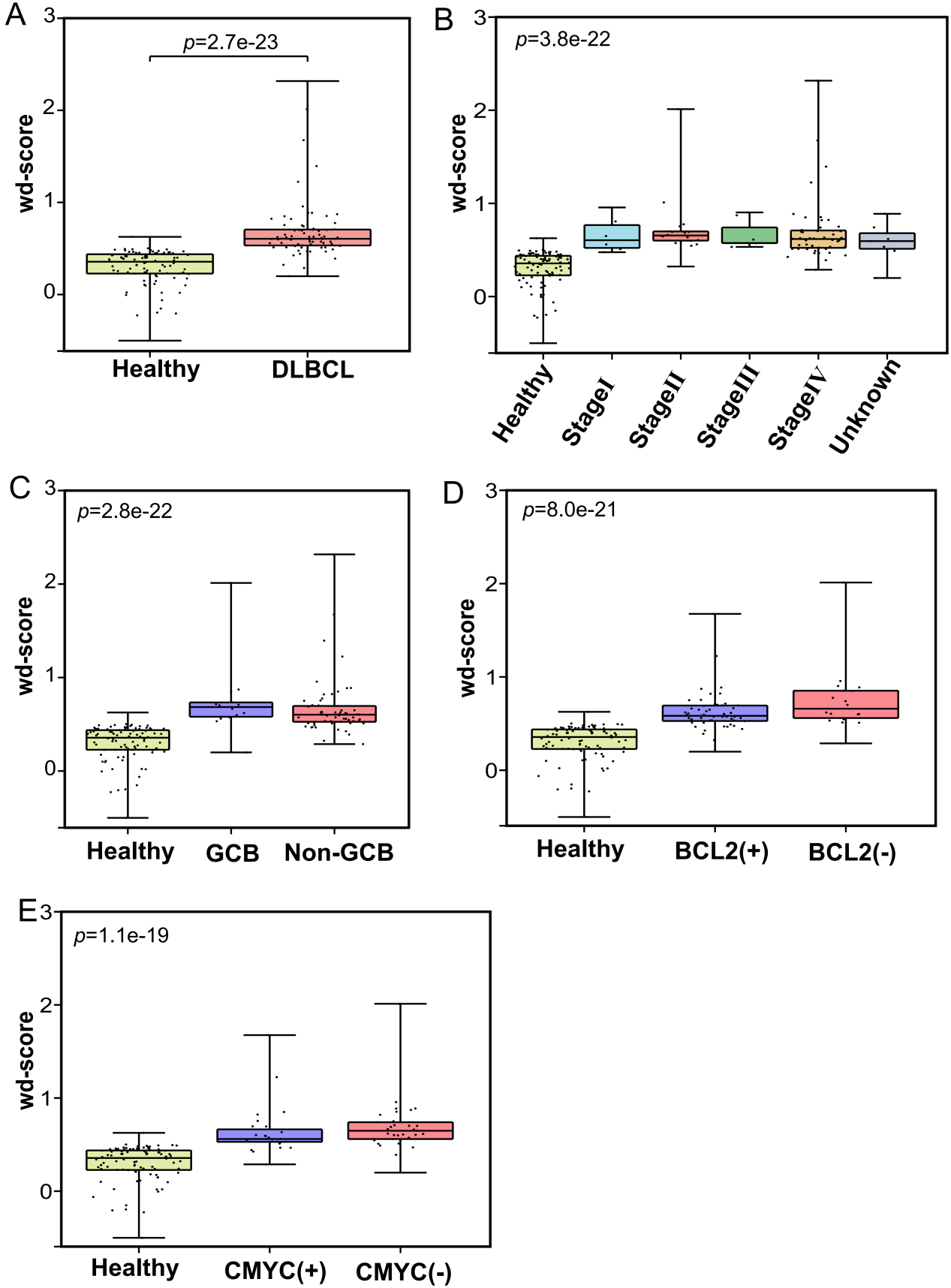


**Supplementary Figure 3. The wd-scores information. A,** Boxplot of wd-score deriving from the integrated model for the DLBCL samples and the healthy controls. **B-E,** box plot of different stages of the disease and clinical diagnostic indicators. GCB: Germinal Center B-cell-like, Non-GCB: Non-Germinal Center B-cell-like.


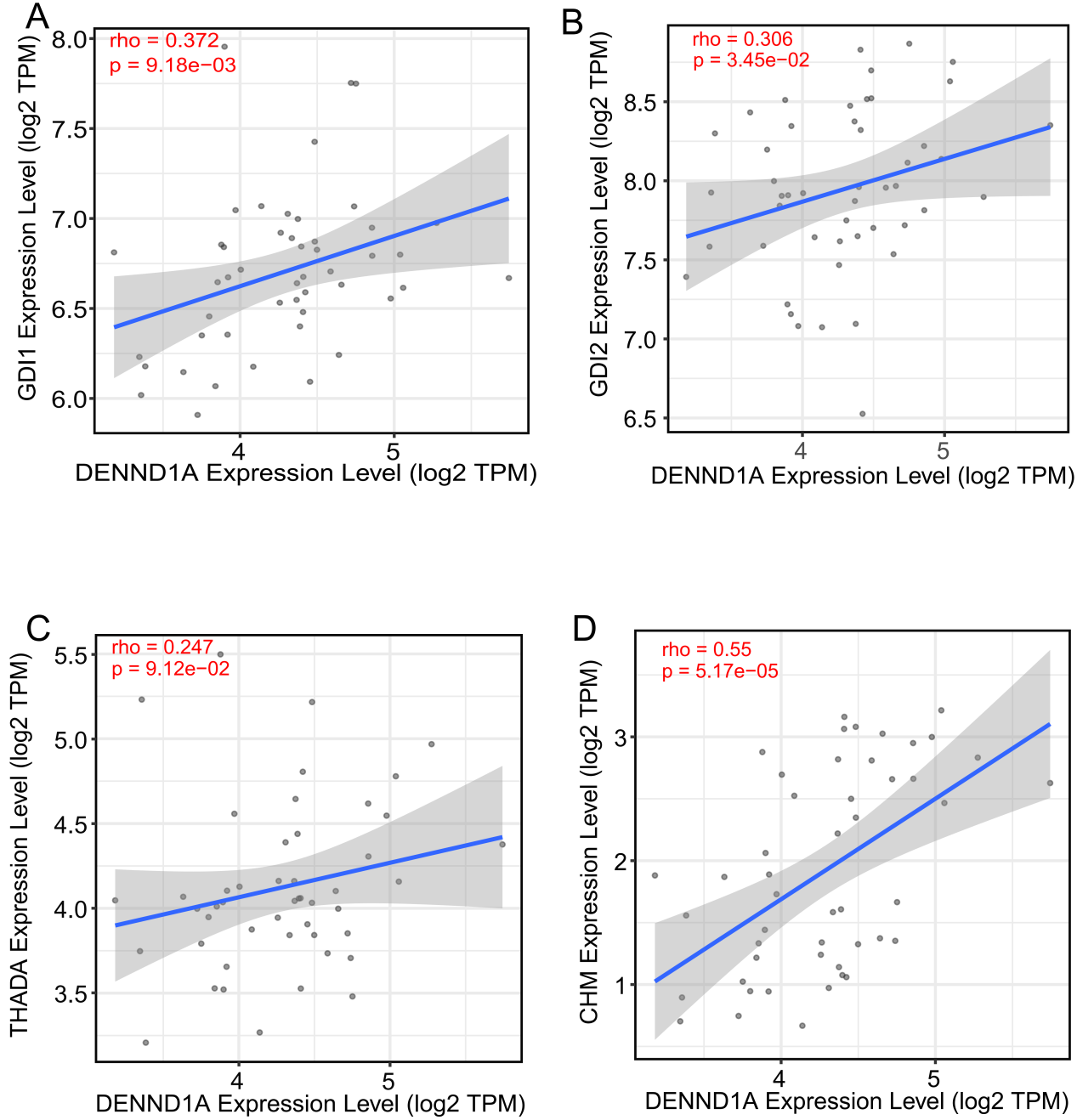


**Supplementary Figure 4. Correlation plots of the mRNA expression of *DENND1A* with the mRNA expressions of *GDI1, GDI2, THADA* and *CHM* in DLBCL in the TCGA-DLBC dataset.** **A,** *GDI1*. **B,** *GDI2*. **C,** *THADA*. **D,** *CHM*.
